# Sleep Quality in the Indian Adult Population During the COVID-19 Pandemic

**DOI:** 10.7759/cureus.17535

**Published:** 2021-08-29

**Authors:** Sukriti Banthiya, Sucheta Sharma, Divya Jahagirdar, Vinay Jahagirdar, Manisha Garg, H.K Sahadev

**Affiliations:** 1 Internal Medicine, Dr. B.R. Ambedkar Medical College, Bangalore, IND; 2 Internal Medicine, Punjab Institute of Medical Sciences, Jalandhar, IND; 3 Internal Medicine, Government Dental College & Hospital, Telangana, IND; 4 Medical Education, Gandhi Hospital and Medical College, Secunderabad, IND; 5 Internal Medicine, Ascension Providence Hospital, Southfield, USA

**Keywords:** covid-19, sleep quality, pandemic, lockdown, general population

## Abstract

Background: In response to the COVID-19 pandemic, social distancing measures such as stay-at-home orders were implemented for all non-essential workers. The consequent disruption in the defined daily work routine has impacted both the quality and duration of sleep. Our aim was to evaluate the quality of sleep in the Indian adult population during the COVID-19 pandemic.

Methods: The data were collected between April 17, 2020 and May 24, 2020, and participants were invited openly through social media platforms (Facebook, Twitter, WhatsApp, Instagram). Sleep quality was evaluated using the Pittsburgh Sleep Quality Index (PSQI) Questionnaire.

Results: The study population consisted of 808 participants (mean age 30.85 years, 56.7% female). The mean sleep score of the study population was 6.78 ± 3.19 on the PSQI, with a majority (57.2%) of respondents showing 'poor' sleep quality (>5 on PSQI). The mean sleep duration of the study population was found to be 6.9 ± 1.4 h, and sleep latency was 42.64 ± 51.6 min. The PSQI scores were comparable for age, gender, and work status and were not significant. However, a significant association between self-reported mental health and quality of sleep was found (p<0.05). Participants who reported a deterioration in mental health were more likely to have poor sleep quality than those who reported an improvement in their mental health.

Conclusions: The results of this study show that poor sleep quality is widely prevalent among the the general population in India during the COVID-19 pandemic.

## Introduction

In December 2019, there was a cluster of pneumonia cases in the city of Wuhan, China [1]. Some of the first cases had reported visiting or working in seafood and live animal markets in Wuhan. Investigations found that the disease was caused by a newly discovered species of the Coronaviridae family of RNA viruses, severe acute respiratory syndrome coronavirus 2 (SARS-CoV-2). The condition, now known as coronavirus disease 2019 (COVID-19) spread within China and to the rest of the world. On March 11, 2020, the World Health Organization declared the outbreak a pandemic [2].

The first case of COVID-19 in India was reported on January 30, 2020; a medical student who had traveled from Wuhan, China, the then epicenter of COVID-19. To contain the spread of the disease, the state and central government implemented several policies and guidelines. These included travel restrictions and social distancing measures such as stay-at-home orders for all non-essential workers. On March 24, 2020, the Indian government announced a nationwide lockdown [3]. The lockdown was executed in four phases with the final stage of the nationwide lockdown (phase 4) enforced by the National Disaster Management Authority (NDMA) and Government of India till May 31, 2020, following which a cautious and staggered approach was taken to re-open the country in phases [4].

The nationwide lockdown and consequent disruption in the defined daily work routine have led to changes in eating habits, exercise regimes, and mental health which have impacted both the quality and duration of our sleep. Sleep is an essential biologic process that plays a critical role in maintaining not only cognitive but also physical health and optimal functioning of the immune system. Disrupted sleep patterns can cause changes in gene expression [5], release of hormones [6], impact metabolism [6], and circadian rhythm [7]. Studies have shown an association between insomnia and numerous disorders, such as obesity [8], type 2 diabetes mellitus [9], hypertension [10], cardiovascular disease [11], arrhythmias [12], immune disorders [13], and even colorectal cancer [14]. Poor sleep was found to have led to a reduced quality of life [15], affect mood [16], cause emotional distress [17], neurodegenerative changes [18], and loneliness [19]. In children, a lack of sleep was found to impact cognitive functioning in children [20] and increase risk-taking in adolescents [21].

The widespread prevalence of low sleep quality during the COVID-19 pandemic could make the population more vulnerable to infectious agents [22] as well as increase the risk of non-communicable diseases. In this study, we aim to study the prevalence of poor sleep quality in the adult population of India and hypothesize that social distancing measures such as the nationwide lockdown have contributed to a greater prevalence of sleep disturbances.

## Materials and methods

Study design and participants

A cross-sectional, observational study was conducted between April 17, 2020 and May 24, 2020. Convenient and random sampling was used in inviting participants to complete the questionnaire, distributed through social media platforms (Whatsapp, Facebook, Instagram, Twitter). The start date and end date were decided so, to be reflective of the sleep quality during the peak phase of the nationwide lockdown, keeping in mind that the sleep quality of the participants was being assessed over a month using the Pittsburgh Sleep Quality Index (PSQI) questionnaire [23]. The voluntary participants were asked to complete an anonymous self-administered questionnaire through a Google form. It took approximately 10 min to complete the questionnaire. The inclusion criteria were adults, who were able to understand English, had access to the internet, and were residing in India at that time. The responses from the population above 18 years of age and people residing or traveling outside India were excluded.

Measures

Socio-demographic Variables

The socio-demographic variables include age, gender, education level, income, occupation, smoking and alcohol consumption, exercise as well as the working situation.

Sleep Quality Measure

The PSQI-English version [23] is a 19-item self-reported questionnaire that is used to measure sleep disturbances and quality over the past month. The total score comprises seven component scores, which include subjective sleep quality, sleep duration, sleep latency, sleep disturbances, sleep efficiency, daytime dysfunction, and the use of sleeping medication. Scores range from 0 to 21. A higher score represents worse sleeping conditions, while a low score denotes good sleeping conditions. A score of >5 was the cut-off value to indicate poor sleep quality in the Indian population.

Statistical analysis

Statistical analysis was performed with SPSS version 19.0. Descriptive statistics such as mean, range, standard deviation (SD), and percentages were calculated. Chi-square analyses were used to determine the association between different socio-demographic variables, gender, occupation, working situation, mental health, exercise, and sleep quality. For all tests, values of p < 0.05 were considered statistically significant.

## Results

A total of 953 participants were invited to participate in the online survey, 808 of whom fulfilled our inclusion criteria and completed the form, corresponding to a response rate of 84.7%. The majority of our respondents were female (56.7%), with a mean age of 30.85 years. Of the population studied, most were college graduates (55.9%) or had a professional degree (37%) and belonged to the upper socioeconomic class (Table 1). In the study population, 44.6% of respondents were healthcare professionals, 28.3% of respondents reported working from home during the pandemic while some of the participants (25.5%) continued to work outside their home (Table 1).

**Table 1:** Demographic variables.

Demographic	
Mean age (range) years	30.85 (18-90)
Age <30	67.32%
Age =>30	32.68%
Male: Female	350:458
Education	
Professional degree	37%
Graduate/Postgraduate	55.90%
Undergraduate	0.70%
Intermediate/Post high school diploma	4.20%
Middle school	0.20%
Primary school	0.10%
None	0.90%
Occupation	
Self-employed	0.70%
Professional/Semi-professional	33.60%
Healthcare professional	44.60%
Skilled	2.40%
Semi-skilled	0.50%
Unskilled	0.10%
Clerical	1.50%
Pensioner	1.20%
Homemaker	2.10%
Unemployed	4.00%
Student	9.30%
Income (per annum in INR)	
>50,000	71.90%
>25,000-50,000	14.20%
>20,000-25,000	4.10%
>15,000-20,000	2.80%
>10,000-15,000	2.10%
2500-10,000	4.60%
Working conditions	
Working from outside home	25.50%
Working from home	28.30%
Employed, not currently working	11.90%
Homemaker	2.10%
Retired	1.10%
Student	21.40%
Unemployed	9.70%

The mean sleep score of the study population was 6.78 ± 3.19 on the PSQI [23], with a majority (57.2%) of respondents showing "poor" sleep quality (>5 on PSQI). The mean sleep duration of the study population was found to be 6.9 ± 1.4 h, and sleep latency was 42.64 ± 51.6 min (Table 2). Almost a third of the respondents (30.7%) stated they had trouble falling asleep three or more times during the past month. "Not being able to sleep within 30 minutes" was cited by the respondents as being the most common cause of trouble falling asleep, followed by "waking up in the middle of the night/morning." Surprisingly, more than half of the study population (58.5%) described the quality of their sleep as "fairly good". Only a small percentage of the study population reported purchasing over-the-counter (OTC) sleep medications over the past month (8.4%) (Table 2). In the same vein, the majority of respondents did not report any trouble doing their daily chores or engaging in social activities (57.5%), or did they have any problems keeping up their enthusiasm throughout the day (59.6%) (Table 2).

**Table 2:** Results of PSQI. PSQI, Pittsburgh Sleep Quality Index {23]

	Results
Average time to go to sleep (min)	42.64 +/-51.62
Average duration of actual sleep (h)	6.92 +/-1.40
Subjective sleep quality	
Very good	18.60%
Fairly good	59.10%
Fairly bad	20.30%
Very bad	3.70%
Daytime dysfunction	42.50%
Mean global PSQI score	6.48 +/- 3.19

The majority of the study participants were health-conscious, continuing to engage in physical exercise (70.3%) during the lockdown. Only a small proportion of the study population smoked cigarettes during the lockdown (57 participants, 7.05%) or consumed alcohol during in the past one month (127, 15.7%) and 241 people (29.8%) reported their mental health had changed for the worse during the past one month.

The PSQI scores were comparable for age, gender, and work status and were not significant. There was, however, a significant association between self-reported mental health and quality of sleep (p < 0.05). Participants who reported a deterioration in mental health were more likely to have poor sleep quality than those who reported an improvement in their mental health.

## Discussion

To the best of our knowledge, this is the first study to assess sleep quality in the general population in India during the COVID-19 pandemic. We used a validated questionnaire, the PSQI, a subjective measure of sleep quality, which differentiates between "good" and "poor" sleep quality by measuring seven domains of sleep. Our participants include only those that had access to the Internet and could read English, as the questionnaire was circulated on various social media platforms in English only. We were not able to establish baseline PSQI scores of the participants before the nationwide lockdown. Limitations notwithstanding, our results show four key findings, especially in the context of studies done before the pandemic that assess sleep quality in the general population.

First, the average PSQI score was 6.48 +/- 3.19, and 57. 1% of the participants were found to have poor sleep quality (PSQI score >5). In contrast, studies conducted in ordinary times showed only 20.4%, and 31.3% of the general population had poor sleep quality [24-25]. Following the trends of the PSQI scores through time showed that sleep quality was the worst during the second phase of the lockdown in India with an average PSQI score of 7.15. The average PSQI scores during phase 1 and 3 of the lockdown in India was 6.17 and 6.34, respectively. Similarly, longer sleep latency was reported by this study cohort during the pandemic as compared to before, although the average duration of sleep remained unchanged [22].

Second, sleep disturbances were noted in 61.8% of our study cohort; a two-fold increase compared to 34.2% of the adult Indian population reported in ordinary times [24]. The perceived sleep disturbances were attributed to two main reasons -- difficulty falling asleep and waking up in the middle of the night/morning. These findings suggest that the pandemic and consequent stay-at-home orders have resulted in poor sleep quality and include problems such as falling asleep and staying asleep despite an unchanged sleep duration.

Third, sleep quality did not differ by age, gender, occupation, level of education, or income. There was no association of quality of sleep with working conditions (work from home vs working outside at their workplace). However, a significant association was found between perceived change in mental health during the pandemic and sleep quality (p < 0.05). Poor sleep quality was found to be more prevalent in those that reported change in their mental health for the worse than those that reported it to be unchanged/changed for the better. One study that assessed the psychological impact of the COVID-19 pandemic in 507 people in West Bengal found that a majority of their respondents (69.6%) were worried about the financial loss they incurred during the lockdown [26]. The study also showed that one-fourth (25.6%) of the respondents found that the COVID-19 pandemic had threatened their existence and one-third (30.8%) found it difficult to adjust to the new routine during the 21-day lockdown period [26]. Given the association between mental health and sleep [26], the prevalence of psychological distress and inability to adjust to the lockdown initially could help explain the trends of the PSQI scores through the four phases of lockdown.

Fourth, 8.4% of our study cohort reported using over-the-counter (OTC) sleep medication during the pandemic, as compared to 1.1% in ordinary times [24]. This suggests the medical community must urgently address sleep behavior and disorder during the pandemic to inform and educate the public on the safe use of OTC for insomnia as well as the non-pharmacological treatments for the same.

To summarize, our study showed that the prevalence of poor sleep quality in the Indian adult population increased during the pandemic as compared to ordinary times, and problems included difficulty initiating and maintaining sleep. A significant association between perceived change in mental health for the worse and poor sleep quality suggests that mental health and sleep quality go hand in hand and must be addressed simultaneously.

Limitations

The results of the study are to be viewed in light of its limitations. Since sample collection was through a self-administered questionnaire the responses were mainly subjective rather than objective which impacts the validity of our results to a certain degree. The method of sample collection applied to the individuals who understand English and had access to the Internet, which creates a possibility of selection bias in the study population. Since the study was conducted in the immediate aftermath of the beginning of the pandemic, the changes in sleep quality of the people were reported to be significantly affected. The responses would have varied greatly, had the data been gathered during the second wave of the pandemic, which implies the need for further studies to observe the changes in sleep quality in the later phases of the COVID-19 lockdown.

## Conclusions

The results of this study show poor sleep quality is widely prevalent among the adult population in India during the COVID-19 pandemic. The lack of good sleep quality could result in increased susceptibility to respiratory infections due to sub-optimal functioning of the immune system. This is of grave concern considering the ongoing pandemic. Reduced sleep quality could also activate a domino effect by adding to the problem of diabetes mellitus and hypertension in the country and subsequently burdening the healthcare system. Closer to home, poor sleep quality will affect mood, productivity, and ability to cope with the shelter in place and social distancing measures.
